# Geospatial Clustering of Opioid-Related Emergency Medical Services Runs for Public Deployment of Naloxone

**DOI:** 10.5811/westjem.2018.4.37054

**Published:** 2018-05-15

**Authors:** Daniel A. Dworkis, Scott G. Weiner, Vincent T. Liao, Danielle Rabickow, Scott A. Goldberg

**Affiliations:** * Keck School of Medicine of University of Southern California, Department of Emergency Medicine, Los Angeles, California; † The Lever Institute, Los Angeles, California; ‡ Brigham and Women’s Hospital, Department of Emergency Medicine, Boston, Massachusetts; § Harvard Medical School, Department of Emergency Medicine, Boston, Massachusetts; ¶ Professional Ambulance, Cambridge, Massachusetts

## Abstract

**Introduction:**

The epidemic of opioid use disorder and opioid overdose carries extensive morbidity and mortality and necessitates a multi-pronged, community-level response. Bystander administration of the opioid overdose antidote naloxone is effective, but it is not universally available and requires consistent effort on the part of citizens to proactively carry naloxone. An alternate approach would be to position naloxone kits where they are most needed in a community, in a manner analogous to automated external defibrillators. We hypothesized that opioid overdoses would show geospatial clustering within a community, leading to potential target sites for such publicly deployed naloxone (PDN).

**Methods:**

We performed a retrospective chart review of 700 emergency medical service (EMS) runs that involved opioid overdose or naloxone administration in Cambridge, Massachusetts, between October 16, 2016 and May 10, 2017. We used geospatial analysis to examine for clustering in general, and to identify specific clusters amenable to PDN sites.

**Results:**

Opioid-related emergency medical services (EMS) runs in Cambridge, Massachusetts (MA), exhibit significant geospatial clustering, and we identified three clusters of opioid-related EMS runs in Cambridge, MA, with distinct characteristics. Models of PDN sites at these clusters show that approximately 40% of all opioid-related EMS runs in Cambridge, MA, would be accessible within 200 meters of PDN sites placed at cluster centroids.

**Conclusion:**

Identifying clusters of opioid-related EMS runs within a community may help to improve community coverage of naloxone, and strongly suggests that PDN could be a useful adjunct to bystander-administered naloxone in stemming the tide of opioid-related death.

## INTRODUCTION

Opioid-associated overdose and death continues at epidemic levels throughout the United States (U.S.) with mortality from opioid use the leading cause of accidental death in the U.S.^1^, ^2^ In Massachusetts (MA) there were 1,990 confirmed opioid-related deaths in 2016, an all-time high and a 19% year-to-year increase over 2015.^3^ Naloxone, a pure competitive antagonist at the opioid receptor, is capable of temporarily reversing the effects of an opioid overdose, and distribution of naloxone to people at risk of opioid overdose as well as their families and friends is a cornerstone of the response to the opioid epidemic.^4^, ^5^ Bystander naloxone, administered by the non-medically trained lay public, has been shown to reverse opioid overdoses and save lives; however, it requires an individual carrying naloxone at the same place and time as an overdose occurs.^2^, ^6^ Efforts to improve community prevalence of naloxone have focused on increasing prescribing and improving availability in pharmacies, and naloxone is now available in many areas either as an over-the-counter substance or under a standing order.^7^ However, barriers to obtaining and carrying bystander naloxone still exist, and bystander naloxone is not currently available everywhere it is needed.^8^–^10^

Unlike bystander-carried naloxone, the public deployment of automated external defibrillators (AEDs) in pre-determined, easy-to-access locations for use by bystanders in cases of witnessed arrest requires no single individual in particular to obtain or carry the life-saving device and shifts the burden of providing potentially life-saving equipment from individuals to the community.^11^, ^12^ Traditionally deployed in settings of high traffic and mass gatherings such as airports, casinos, or sports stadiums, distribution of AEDs has recently been guided by geospatial analyses of cardiac arrest data and pedestrian traffic with encouraging results.^13^, ^14^ Publicly deploying naloxone in AED-like kits may improve naloxone availability to overdose victims and overcome barriers associated with current bystander-carry methods. However, like AEDs and cardiac arrests, determining where to place potential PDN kits requires understanding where opioid overdoses occur. Recent work by our team and others has shown spatial clustering of opioid-related emergency department visits, opioid-related deaths, and self-reported bystander naloxone use, suggesting that opioid overdoses may also show spatial clustering amenable to PDN placement.^15^–^17^

We performed a geospatial analysis of emergency medical services (EMS) runs involving suspected or confirmed opioid overdose in the community of Cambridge, MA. We hypothesized that opioid overdoses do not occur randomly but instead show spatial clustering, and that identifying these clusters would both support the concept of publicly deployed naloxone and help identify locations where naloxone could be stationed for maximum potential effect.

## METHODS

### Study Design and Selection of Participants

This was a retrospective analysis of EMS runs that occurred in Cambridge, MA, between October 16, 2016 and May 10, 2017. Cambridge, MA, is a community of approximately 110,000 citizens spread across approximately 17 Km^2^; EMS calls in Cambridge, MA, are served by a public-private partnership using the public fire service and a single, private EMS company, ProEMS.^18^, ^19^ As part of their standard operating procedures, EMS providers record pertinent information in an electronic medical record maintained by the EMS service. All runs for which overdose is part of the dispatch information or provider impression, or in which naloxone was administered by bystanders, first responders or EMS, are submitted to the Cambridge Department of Public Health. All cases, including the EMS patient care record and narrative were reviewed by an independent epidemiologist blinded to our study hypothesis. We included in this analysis any case for which the Cambridge Department of Public Health determined a suspected or probable opioid overdose. Data were manually reviewed for duplicate entries. We excluded any runs originating outside of Cambridge, MA. All data manipulation and statistical analysis was performed using the R programming language.^20^ This study was approved by the institutional review board at Partners Healthcare Boston, MA.

**Population Health Research Capsule**

What do we already know about this issue?

Acute opioid overdose can be reversed by bystanders using naloxone, but only if they have access to it. Deploying naloxone kits in set locations might improve public access and facilitate overdose reversal.

What was the research question?

Do opioid overdose-related emergency medical services (EMS) runs in Cambridge, Massachusetts, show distinct geospatial clusters where naloxone might be deployed?

What was the major finding of the study?

Among 700 EMS runs, we found significant geospatial clustering with approximately 40% occurring within 200 meters of one of three distinct hot spots.

How does this improve population health?

Identifying clusters of opioid-related EMS runs within a community may help to improve community coverage of naloxone by suggesting specific locations for publicly deployed naloxone kits.

### Geocoding EMS Runs

Geocoding is the process of determining the exact spatial location of an address: During this process, a human-readable address such as 795 Massachusetts Ave, Cambridge, MA 02139 (Cambridge City Hall) is transformed to spatial coordinates (e.g., X: −71.106026, Y: 42.36681), which are amenable to mapping and statistical analysis. We performed first-pass geocoding of addresses of EMS runs using the U.S. Census Geocoder and address-batch geography lookups matched to U.S. Census 2010 data vintage, which provided coordinates in the North American Datum 1983 (NAD83).^21^ Addresses not successfully geocoded by the U.S. census were geocoded using Google Maps, which provided coordinates in the World Geodetic System (WGS84).^22^ NAD83 and WGS84 systems are equivalent to within approximately two meters over the small areas involved in this study, so the two systems were treated interchangeably for all geospatial analyses reported here.^23^–^25^ Cambridge, MA, city boundaries were defined by the Geographic Information System of the City of Cambridge, MA.^26^ Projections between latitude/longitude (degrees) and Cartesian coordinates (meters [m]) were performed using “sp” package in R.^27^ Maps of EMS runs in Cambridge, MA, were produced using either the “spatstat” package in R, or QGIS software with base maps provided by OpenStreetMaps.^28^–^30^

### Geospatial Analysis

Analysis of global spatial clustering of EMS runs, asking the question – do EMS runs cluster at all in Cambridge, MA? – was examined through calculations of Ripley’s K-function [K(r)]. We performed calculations of Ripley’s K function, as well as Monte Carlo estimates (MCE) of expected envelopes of K(r), using the “spatstat” package in R with Ripley’s isotropic corrections at window borders.^28^ Briefly, K(r) tests for clustering in a pattern of spatial points by examining observed vs. expected distributions of points around an index point within circles of various areas; in the setting of complete spatial randomness, the density of points should be uniform, so the expected number of points scales with the area of the test circle and should produce an exponential plot of K(r) vs. the circle radius. Compared to the global analysis of clustering provided by the Ripley’s K function, local analysis of clustering addressing the question of where exactly within Cambridge, MA, clusters might occur was performed using density-based clustering. We used an unsupervised, spatial density-based clustering algorithm – the density-based spatial clustering of applications with noise (DBSCAN) method via the “dbscan” package in R, after projecting coordinate data to the European Petroleum Survey Group (EPSG) Projection 26986.^31^, ^32^ Epsilon neighborhood parameters for the DBSCAN algorithm (EPS) was estimated at 200 using visual inspection of k-nearest-neighbor (KNN) plots (Supplemental Figure 1) with minimum KNN cluster sizes set to three members as described in the DBSCAN vignette.^31^ To maximize the potential utility of identified clusters, we only considered clusters of opioid-related EMS runs with at least 69 runs (10% of the total number of successfully geocoded runs in Cambridge, MA). We calculated distributions of distances between cluster points and cluster centroids in the EPSG 26986 projection using the “raster” package in R.^33^

## RESULTS

### Characteristics of EMS Runs

**Figure 1:**
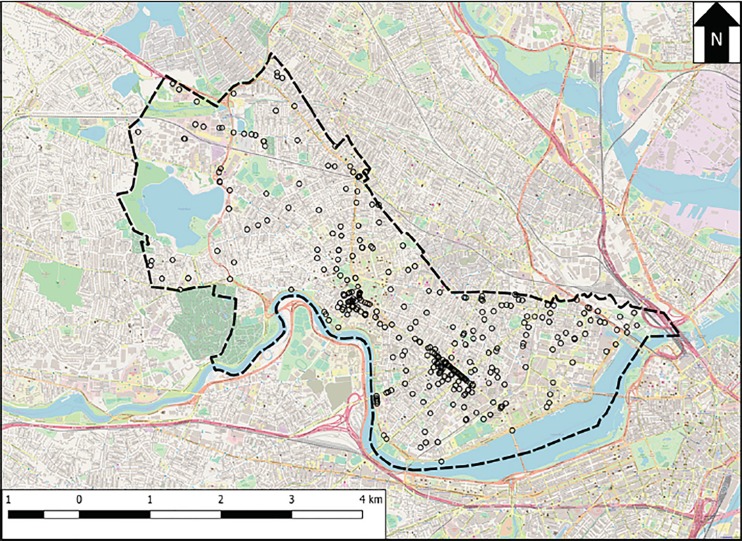
Map of locations of opioid-related emergency medical services (EMS) runs in Cambridge, Massachusetts (MA). Open circles represent locations at which at least one EMS run occurred during the study period. The dashed line shows the border of the city of Cambridge, MA. A scale bar is provided in the bottom left, and the arrow labeled “N” at the top right points due north. Background map data, obtained from OpenStreetMap contributors, is available at www.openstreetmap.org.

Between October 16, 2016, and May 10, 2017, we identified 700 opioid-related EMS runs in the ProEMS database, spread among 359 unique addresses in Cambridge, MA. Of these addresses, 353 (98.3%) were successfully geocoded to 349 unique physical locations; the majority (327, 92.6%) were geocoded using the U.S. census, and an additional 26 addresses (7.4%) were geocoded using Google Maps. The discrepancy between address and physical locations reflects the fact that multiple distinct addresses can occur at the same coordinates, such as with a multi-unit apartment building. For the remainder of our analyses, we used a location-based, as opposed to an address-based, approach. Collectively, these 349 locations accounted for 693 (99.0%) of the initially identified 700 runs. Figure 1 shows a map of the locations of EMS runs in Cambridge, MA, during the study period. Of note during mapping, three locations (each with one run) were found to lie outside the official spatial boundary of Cambridge, MA, and were removed from further analyses, resulting in a final dataset of 690 geocoded runs. Of these 690 runs, we recorded information on patient gender for 683 runs (99.0%), and patient date of birth for 677 runs (98.1%); patients ranged from less than one year of age to 107 years old at the date of EMS service, with a median age of 36 years (interquartile range ([QR] 29–49 years), and the majority were male (422, 61.8%).

### Geospatial Clustering

**Figure 2:**
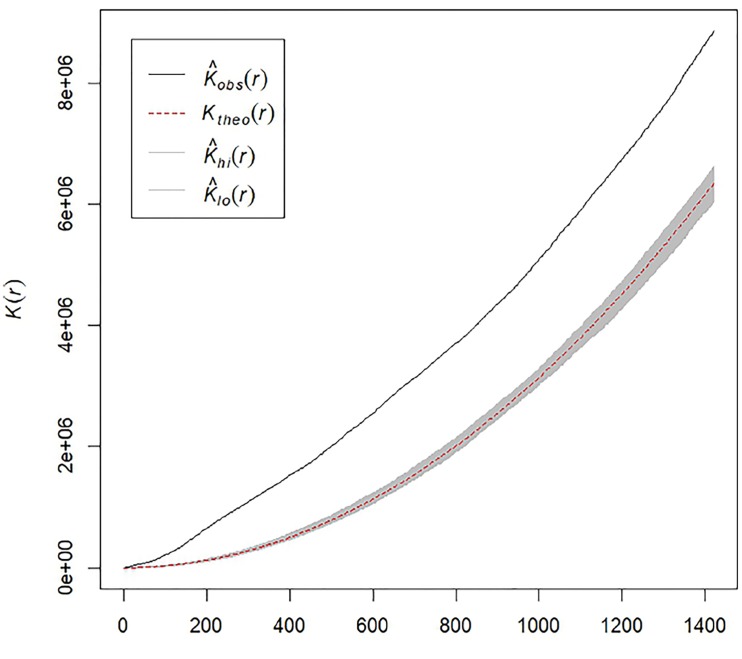
Estimates of Ripley’s K-function (K) for opioid-related runs. Monte Carlo estimates (MCE) of observed vs. expected values of Ripley’s K-function as a function of distance (r). The solid black line shows the estimated observed K(r), while the dashed red line shows the theoretical K(r) in the setting of complete spatial randomness for the same number of observations. The gray-shaded area shows estimates of potential variability in K(r) assuming complete spatial randomness, generated by MCE with n=999 simulations. *Obs*, observed; *Theo*, theoretical; *Hi*, Maximum MCE of theoretical distribution of K(r); *Lo*, minimum MCE of theoretical distribution of K(r).

To test the hypothesis that opioid-related EMS runs in Cambridge, MA, show spatial clustering, we estimated Ripley’s K-function for the set of 690 EMS runs that were geocoded within Cambridge, MA. Figure 2 shows an estimate of the K(r) function for the observed EMS runs, as well as a theoretically expected envelope generated by a MCE with 999 simulations of completely random spatial distributions of EMS runs within Cambridge, MA. As the observed estimate of K(r) deviates substantially from the MCE-generated expected envelope at multiple radii, there is statistically significant evidence of EMS runs clustering with an MCE approximate p-value of p ~ 0.001.

While computing K(r) shows evidence that opioid-related EMS runs do cluster in general across the study area, understanding where to optimally place PDN sites would require more granular knowledge on locations of individual clusters within the study area. To begin to look for these clusters, we first searched for evidence of clusters of opioid-related EMS runs occurring at an individual location. Of the 346 unique locations in Cambridge, MA, 242 locations (69.9%) had a single run each; 103 locations (29.8%) had between two and 16 runs each, collectively accounting for 372 runs (53.9%); finally, a single outlier location had 76 EMS runs, individually accounting for 11.0% of all runs during the study period. This outlier location is a community-based service organization that provides recovery services and emergency shelter to homeless individuals, including those struggling with drug and alcohol addiction.^34^ Compared to runs originating at other addresses, EMS runs originating at this emergency shelter involved patients who were older, with a median age of 43 years (IQR 36.5–58.5 years) for patients coming from the emergency shelter compared to 35 years (IQR 28–48 years) for patients coming from elsewhere in Cambridge, MA. No significant differences were observed in patient gender between patients coming from the service organization or from elsewhere in Cambridge, MA.

**Figure 3:**
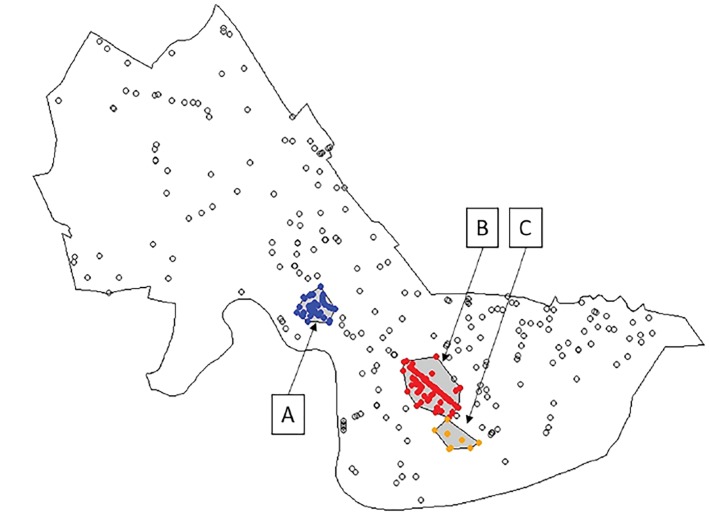
Density-based clustering of opioid-related emergency medical services (EMS) runs. Map of locations of opioid-related EMS runs with superimposed cluster analysis. Filled and unfilled circles both identify locations at which at least one EMS run occurred during the study period. Unfilled circles show locations not in clusters, while filled circles show locations in clusters and are colored by cluster membership. The areas encompassed by identified clusters are shaded in gray. The outer black line shows the boundary of Cambridge, Massachusetts, while the inner black lines surrounding clusters show the convex hull polygons enclosing each cluster. Labels “A,” “B,” and “C” identify and name the clusters.

After identifying this single-location cluster, we next considered clusters of EMS runs that spanned multiple, distinct locations, using an unsupervised, density-based clustering approach. Figure 3 shows the three distinct clusters of opioid-related EMS runs identified, named clusters “A,” “B,” and “C.” Collectively, 362 EMS runs (52.5%) were located in one of the three clusters. Cluster A includes 86 EMS runs (12.5%) from 42 separate locations covering a roughly circular area of approximately 116,948m^2^ (0.05miles^2^) centered on the Harvard Square area, a busy, mixed commercial-residential area containing a public transportation hub and parts of Harvard University. Cluster B was the largest cluster, involving 191 EMS runs (27.7%) from 81 separate locations spread over a linear/ellipsoid area covering approximately 319,630m^2^ (0.12miles^2^) along Massachusetts Avenue at the Central Square area, another large, mixed commercial-residential area containing a public transport hub. Finally, Cluster C included 85 EMS runs (12.3%) from only eight separate locations, one of which is the single-location cluster previously identified, which accounted for 76 (89.4%) of the EMS runs in Cluster C. The table summarizes geospatial and run-related details about these three clusters.

### Modeling PDN Sites

**Figure 4:**
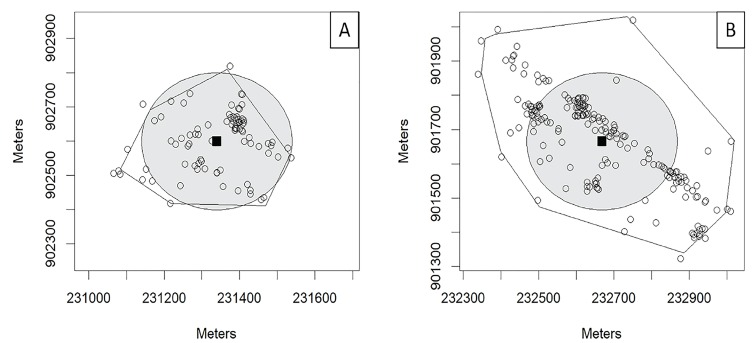
Publicly deployed naloxone coverage areas in opioid-related emergency medical services (EMS) run clusters. Sub-maps of locations of opioid-related EMS runs in Cambridge, Massachusetts, centered on Cluster A (left) or Cluster B (right). Open circles represent locations in each cluster where at least one EMS run occurred during the study period. (A random spatial jitter has been applied to reduce numbers of degenerate points and better show approximate numbers of EMS runs at each location.) Locations of EMS calls that were not part of the relevant cluster are not shown. Solid lines surrounding clusters show the convex hull polygons describing the boundary of each cluster; because of the random spatial jitter, run locations may artificially appear outside of these polygons. Solid squares show the location of the centroid of each cluster, and shaded gray circles show circles with radii of 200 meters centered on cluster centroids.

For clusters A and B, which involved EMS runs spread widely across multiple locations, we modeled the potential impact of sites located at cluster centroids. For the purposes of these models, we assumed the PDN sites to be accessible within 200 meters (m) in any direction of the cluster centroid. This number was chosen to match the epsilon neighborhood parameter of the density-based scan statistic, but is an assumption of the distance a bystander would be willing to travel to access a PDN site. Figure 4 shows maps of Clusters A and B with shaded circles representing the area within which the PDN was modeled to be accessible. For the purposes of visualization, a spatial jitter (random, small, spatial “nudge”) was applied to EMS runs in these clusters to better show call volumes at different locations in each cluster by allowing runs occurring at the same location to be displayed simultaneously on the map. With these assumptions, PDN sites at cluster centroids could potentially have modified 75 EMS runs (87.2%) from Cluster A, and 116 EMS runs (60.7%) from Cluster B. Cluster C involves 85 runs, but 76 of these runs come from a single location – assuming a PDN site was placed inside the service organization at that single location, and that all 76 EMS runs from this individual site might be modifiable by a PDN site at that location, deploying PDN across all three clusters could potentially have modified 267 EMS runs, 38.7% of the total runs during the study period.

## DISCUSSION

We found that EMS runs involving opioid overdose exhibit geospatial clustering in Cambridge, MA, and identified three distinct geospatial clusters as potential targets for publicly deploying naloxone. To our knowledge, this is the first work to examine spatial clustering of opioid overdoses at the level of spatial granularity required to pinpoint potential sites of naloxone deployment. Our findings show two distinct types of spatial clusters, which may require different methods of naloxone deployment: clusters “A” and “B” are both centered at highly trafficked public areas in Cambridge, MA, while cluster “C” represents a spike of opioid-overdoses occurring at a single location.

The optimum strategy for delivering naloxone to Cluster C would likely be locating naloxone kits at or inside the identified emergency shelter. By comparison, there might be multiple strategies for PDN sites within Clusters A and B, the simplest of which would be to position PDN sites at cluster centroids we modeled here. Positioning PDN sites at the cluster centroid is an inherently naïve solution that does not account for geographic realities such as vehicle and pedestrian access to various locations within a cluster, public visibility, and accessibility at off hours. Further work would be needed to understand how to optimize PDN placement within a cluster accounting for these geographic realities, and different clusters likely have different optimal solutions. Still, using the simple models of naloxone deployment at cluster centroids, our results show that approximately 40% of the opioid-overdoses in this dataset would have occurred within 200m of a potential PDN site, suggesting that deploying naloxone at these sites would have a large impact on improving the availability of naloxone where it is needed most.

Beyond simply providing targeting information for stationing naloxone kits, understanding local clustering patterns in opioid-related EMS runs could provide crucial information for a broader, multidisciplinary approach to a community’s response to the opioid epidemic. Knowledge of cluster location and EMS transport patterns could be used to identify potential community partners, for example, large academic centers such as a large university located in cluster A, a second large university located close to Clusters B and C, coalitions of business owners such as those in Clusters A and B, or specific hospitals that handle large portions of EMS transport from particular clusters. Knowledge of cluster locations could also inform other efforts to respond to the opioid epidemic including potentially where to deploy ambulances or where to focus efforts on training first responders and the lay public on bystander naloxone delivery.

While these clusters represent effective potential PDN sites, future work combining these maps with spatial information about public naloxone use, deaths from opioids, or overdoses involving synthetic opioids such as fentanyl or car-fentanyl, could further optimize PDN placement within Cambridge, MA. Similarly, it might be useful to consider other sites of public access to emergency equipment that already exist and compare clusters of opioid-related EMS runs to the locations of AEDs already deployed in Cambridge, MA. Future work is also needed to consider the details of how PDN sites physically would be constructed, how the naloxone would be stored, and how they could be made most easily accessible to the public. In general, geospatial analysis of a particular subset of EMS runs, such as opioid-related runs, could be a useful tool for focusing community engagement, education, and intervention.

## LIMITATIONS

This analysis of geospatial clustering of opioid-related EMS runs is limited to the underlying data captured by Cambridge’s EMS services, and therefore might not include all opioid overdoses in Cambridge, MA. While the total number of opioid overdoses occurring in Cambridge, MA, during the study period is therefore likely greater than the 700 EMS runs we consider here, it is not possible to determine where non-recorded overdoses occur geospatially. While inclusion of EMS runs into our data was determined by a trained epidemiologist independent to our study who examined all EMS data, we did not have access to outcome data including toxicological testing and hospital records. Thus, it was not possible to confirm overdose in each case with certainty. Additionally, the raw data for each run were not available so it was not possible to independently verify the epidemiologist’s assessment. However, these cases do likely represent the patients who would receive naloxone in a PDN program. Collectively, these facts might introduce error into our clustering, which is inherently only as good as the data it is built on. A minor limitation is the inability to independently verify the age of the one patient transported by EMS with a reported age of 107; it is not possible from available data to determine if this patient actually was 107 years old or had a default date of birth of 01/01/1910 entered.

Within each cluster, the percentage of EMS runs that we label as “potentially modifiable” is dependent on our assumption of 200m as a travel distance to a PDN site. As discussed above, optimal placement of PDN sites requires further study, and bystanders might be willing to travel more or less than this distance depending on factors such as the built environment, weather, and time of day. Additionally, the analysis we performed here is limited to a single city served by a single EMS service, and more work would be needed to extend the modeling solution developed here to other cities including cities served by multiple EMS services each with partial data. Specifically, larger cities or cities with unique geographic features such as rivers or geographic boundaries that partition the city would require more robust spatial analysis. Each city considering implementation of PDN sites would need to analyze city-specific overdose data to optimize PDN positioning.

Finally, it is not yet known if placing PDN sites would improve outcomes for cases of opioid overdose or would actually offer a quicker delivery of naloxone over EMS administration when studied in real life, and significant future work would be needed to investigate if this is the case. We believe that this analysis offers the theoretical and geospatial grounding for performing an “*in vivo*” PDN study and determining its utility as a response to the opioid epidemic.

## CONCLUSION

Opioid overdoses show spatial clustering in this geospatial analysis of EMS runs in Cambridge, MA, with three distinct clusters of opioid overdoses identified. In general, public deployment of naloxone in areas of high opioid overdose could be a useful and important adjunct to other methods of naloxone delivery including bystander naloxone and first-responder naloxone. Identifying clusters of opioid-related EMS runs within a community is a key first step.

## Supplementary Information



## Figures and Tables

**Table:** Characteristics of clusters of opioid-related emergency medical services runs.

Cluster	Runs	Locations	Area	M-Dist	N-200	P-200	M-Age	P-Female
A	86	42	116948.3	97.2	75	87.2	38	34.9
B	191	81	319630.7	171.7	116	60.7	37	31.4
C	85	8	94332.4	17.7	80	94.1	40	35.3

Runs: total number of emergency medical services (EMS) runs included in cluster.

Locations: unique spatial locations included in cluster.

Centroid: coordinates of cluster centroids, listed as Latitude/Longitude with WGS84 coordinate reference.

Area: physical size of cluster in square meters.

M-Dist: median distance in meters between all points in a cluster and the centroid of that cluster.

N-200 and P-200: number and percentage of EMS runs in a cluster falling within 200 meters of the cluster centroid.

M-Age: median age in years of patients receiving EMS care within a cluster.

P-Female: percent of patients receiving EMS care within a cluster that was identified as female.
