# Supplementary material for: Geospatial Clustering of Opioid-Related Emergency Medical Services Runs for Public Deployment of Naloxone
Source: West J Emerg Med. 2018 May 15;19(4):641–8. doi: 10.5811/westjem.2018.4.37054 (PMC6040905; doi:10.5811/westjem.2018.4.37054)
Supplement: Supplementary file 1 [file wjem-19-641-s001.docx]

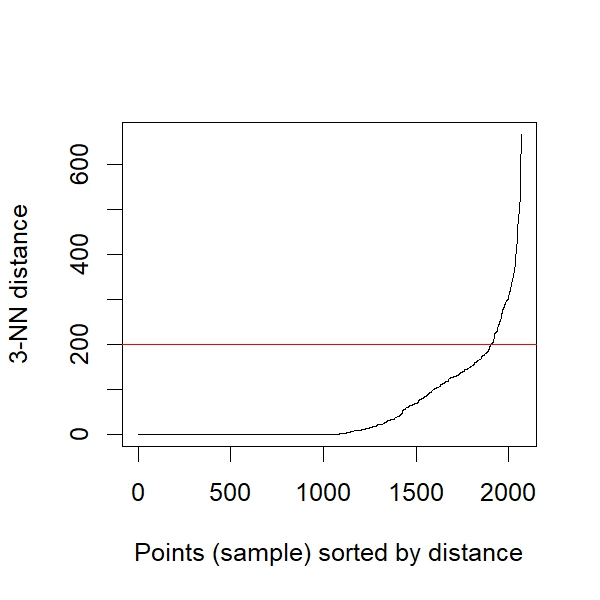


**Supplemental Figure 1.** K-Nearest Neighbor plot of locations of opioid-related EMS runs. Plot of distances of three nearest-neighbors (3-NN) calculated over all opioid-related Emergency Medical Services (EMS) runs. The solid red line at 3-NN distance = 200 meters shows the EPS value used for further DBSCAN calculations. *EPS:* Epsilon neighborhood parameters.
